# A Comprehensive View of Frozen Shoulder: A Mystery Syndrome

**DOI:** 10.3389/fmed.2021.663703

**Published:** 2021-05-11

**Authors:** Daniel de la Serna, Santiago Navarro-Ledesma, Fany Alayón, Elena López, Leo Pruimboom

**Affiliations:** ^1^PNI Europe, The Hague, Netherlands; ^2^Department of Physiotherapy, Faculty of Health Sciences, Universidad of Granada, Melilla, Spain; ^3^Department of Physiotherapy, Universidad Pontificia de Salamanca, Salamanca, Spain

**Keywords:** frozen shoulder, shoulder pain, low grade inflammation, microbiome, chronic hypoxia, fibrosis, insuline resistance, sedentary life style

## Abstract

Frozen shoulder is a common epidemiological affliction. Data acquired from people who suffer from this type of damage in other joints such as the hip, wrist and ankle also exist; although these syndromes are less common. Treatment for frozen shoulder is primarily physical (physiotherapy, manual therapy), secondary medical (corticosteroid injections) and finally surgical but with limited success. The difficulty in treating this type of condition successfully lies in the lack of knowledge about the risk factors involved and the pathophysiology underlying this mysterious syndrome. This review gives an overview of the current scientific position of frozen shoulder in terms of evolutionary factors, etiology, the different mechanisms of action involved, current treatment options and other possible interventions based on recent discoveries of pathophysiological mechanisms. The overall objective is to clarify several unknown aspects of a syndrome that affects up to 5% of the world's population.

## Introduction

Sometimes the shoulder joint becomes stiff, inextensible, and painful in a mysterious way, which is how frozen shoulder (FS) presents itself. Its etiology can be both primary or secondary to other conditions. Although frozen wrist (1), frozen hip (2) and frozen ankle (3) syndromes also exist, FS is the most common “frozen” syndrome (4). It is a difficult condition to define and diagnose precisely, as well as to treat effectively.

Some known risk factors for its development are diabetes, Dupuytren's syndrome, thyroid disease (especially hypothyroidism), nephrolithiasis, cancer, Parkinson's disease, shoulder injury, smoking, heart and neck surgery or chronic regional pain syndrome (5). Comorbidities occur in 85% of patients with FS and 37.5% have more than three comorbidities (6).

FS can be very disabling, and, against the current opinion of many health professionals, FS does not resolve spontaneously in a large number of patients (7). Understanding the shared pathways between FS and the aforementioned comorbidities opens the possibility to develop new interventions based on common pathophysiological mechanisms such as insulin resistance and low-grade inflammation (LGI).

### Epidemiology

The literature indicates that the incidence of FS is 2 to 5% worldwide (8). The prevalence among the diabetic population and those suffering from pathologies of the thyroid gland, especially hypothyroidism may increase as high as 10 to 38% (9). Most people who develop FS do so between 40 and 60 years of age (10), being unusual in patients over 70 with the exception of secondary traumatic FS (11). The incidence among women is 1.6 (12) to 4-fold higher than in men (4). Black/African Americans and Hispanic/Latinos also show a higher prevalence (10). Type 1 diabetes is the greatest risk factor for the development of FS, where the incidence can reach 59% in patients over 45 years of age with a lifetime prevalence of 76%. This group of patients has greater associated disability, along with a greater reduction in their range of mobility (13). The accumulated level of glycated hemoglobin A1c (HbA1c) is a determining factor, hence those patients with poorer blood glycemia control accumulate an increased risk for the development of FS (14).

Finally, incidence is also expressed with significant contralateral differences *p* < 0.01: the non-dominant arm is affected more than the dominant arm (58.9–41.1%) and the left arm more than the right (53.4–46.6%) (15). Interestingly, a hypothesis to explain FS is proposed by the authors of the aforementioned study which states that the pathology could be more related to an asymmetric control of the brain rather than to some traumatic factor. The prognosis of FS is highly variable. In contradiction to the common assumption that FS completely resolves over time, a meta-analysis found this to be scientifically invalid and that if left untreated, FS may persist for the more than 3 years or even never resolve (7).

### Pathogenesis of Frozen Shoulder

Mechanisms that may be key to the development of FS, such as the accumulation of advanced glycation end products (AGE) in the shoulder (16) associated with insulin resistance with compensatory hyperinsulinemia (17), chronic hypoxia (18), chronic LGI (19), and endotoxemia, also exist.

FS is characterized by the spontaneous onset of symptoms such as pain, stiffness and progressive loss of mobility (20). It presents in some cases, but not all, as a fibrotic state with capsular and ligament involvement and an inflammatory-based contracture that restricts the rotational interval of the affected shoulder.

Although agreement on the development of FS is not unified, it seems that it is proliferative, fibroblastic and inflammatory in nature (21). Data from recent studies, using synovial biopsies of patients with FS, have shown a chronic presence of immune cells (mast cells, macrophages, T and B lymphocytes), as well as inflammatory mediators including cytokines, such as interleukins (IL)-1β, IL-6, IL-8, and tumor necrosis factor-alpha (TNF-α) (22), and matrix metalloproteinases (MMPs) (23) in the affected shoulder. These pathological mechanisms appear to be responsible for the activation of fibroblast and deregulation in collagen synthesis in people suffering from FS.

Another study showed the presence of S100 in 77% of the research patient group suffering from FS (*n* = 22, 17 of 22). S100 is a biomarker for the presence of neuronal tissue and the increased presence of this nerve tissue could give a partial explanation for the pain associated with FS (24).

The evaluation of cadavers and the use of open arthroscopic surgery have shown synovial hyperplasia with increased vascularization during the initial period (25). It ends with an incipient fibrosis located mainly in the rotator cuff interval (formed by the tendons of the subscapular, biceps and supraspinatus muscles, and coracohumeral and superior glenohumeral ligaments), together with the base of the coracoid process (26) and in the subscapular recess (27).

The thickening of the coracohumeral ligament, which is the roof of the rotator cuff interval, is indicated as one of the specific manifestations of FS and the main limiting factor for external rotation, although given its relationship with the subscapular and supraspinatus tendons it also contributes to restricting internal rotation (28). In more advanced stages of FS, the thickening and contraction of the glenohumeral capsule culminates in limiting the range of movement in all directions (25).

Recently, two studies in a subgroup of patients diagnosed with FS have shown that there is no significant capsule restriction (29, 30). In a sample of five patients with an indication for capsulotomy (loss of active and passive mobility >40% in at least two planes of movement in the affected shoulder relative to the healthy one), it was found that under general anesthesia, the movement of the affected shoulder was less restricted. All patients showed a significant increase in their passive range of motion (ROM), especially in abduction while under general anesthesia conditions, compared to their ROM while awake. The subject with the smallest improvement achieved an increase of 44° in abduction under anesthesia (94° awake and 138° under general anesthesia), while the subject with the greatest improvement increased abduction by 83° under general anesthesia (55° awake and 138° under general anesthesia) (29).

In a more recent study (30), the above research group confirmed the results of their study carried out in 2015 with five more patients suffering from FS and presenting with significant loss of shoulder range of motion in abduction and external rotation. Hollman L et al. again found that under general anesthesia the range of motion improved in both abduction, 55° (from 55° awake to 110° under general anesthesia) in all patients, and in external rotation, 25° (from 15° awake to 40° under general anesthesia) in three patients (30). This finding supports the hypotheses of the existence of a fear-based brain induced movement limitation, although more research is needed to determine the number of patients suffering from this type of FS.

The cited data help to better understand the development of FS and show that progression has been made in regard to the knowledge of the possible risk factors and the underlying pathophysiological mechanisms causing FS. Although this advance is clear, FS is still not completely understood and epidemiological data show the need for further investigation (4). The purpose of the review is to increase knowledge about less known risk factors and pathophysiological mechanisms which lead to the development of FS and also to propose new interventions based on those factors and mechanisms.

### Frozen Shoulder and Its Possible Evolutionary Origin

Bipedalism liberated the hands which made it easier for humans to throw projectiles. The human shoulder evolved by optimizing the storage and generation of elastic energy, especially in the maximum range of external rotational movement, which facilitated the launching of projectiles at high speed (31). From an evolutionary perspective, manual work and the use of the upper limbs, especially among women, used to be much higher than today (32). Today's lifestyle has dramatically decreased the use of the upper limbs, in contrast to its evolutionary design, most likely leading to atrophy of parts of the complex ligament capsule of the shoulder (19).

The non-use of a body part can result in neuroanatomical reorganization in different parts of the brain, such as occurs with phantom pain (33) or in the production of pain or neglect syndrome of that same part (34). The fear of pain or the fact that little used tissue can be more easily damaged can produce a fear-based brain reaction and generate a muscle defense response that could lead to FS (30, 34).

The shoulder, the joint in the body with the greatest range of motion, would have its movement further limited by this possible muscle protection response (35). Lack of movement may be responsible for chronic hypoxia in a joint that already has low partial oxygen pressure (pO2) (36), providing a suitable environment for the establishment of an inflammatory process mediated by the activation of transcription factors such as hypoxia- inducible factor 1 (HIF-1), nuclear factor Kappa B (NF-kB) as well as the activation of several vascular and endothelial growth factors (VEGF) and MMP 1, 3 and 13 (36) which are associated with inflammation, angiogenesis and tissue destruction (37).

A sedentary lifestyle is a risk factor which does not depend on the physical activity performed; it is also associated with an increase in inflammatory activity and the development of insulin resistance (38).

Similarly, oxidative stress associated with the presence of inflammatory cytokines affecting different parts of the shoulder ligament capsule complex may lead to an increased accumulation of free radicals, advanced glycation end products and, probably, subclinical alterations in both the connective tissue and the extracellular matrix (19). The fact that the most affected shoulder is the non-dominant one, commonly the left one (15) may be indirect evidence that part of the pathophysiology of FS is associated with a lack of movement and weakening of the different joint tissues (ligaments, capsule, synovial sheath and rotator cuff) as a result of “cerebral” joint immobilization and associated muscle defense. Further proof that FS could be caused by a lack of mobility associated with a neglect and/or muscular defense is the fact that people who have suffered from hemiplegia have a higher risk of suffering FS (39). A neglect syndrome may also be significant evidence of having had hemiplegia and could be the explanation for the risk of presenting FS after a stroke (40). In short, it seems that the underuse of the full range of motion in modern sedentary humans could cause a predisposition for the development of FS. Other more recent risk factors would then exacerbate the actual syndrome called FS.

### Chronic LGI, the Role of Alarmins, Mechanical Stress, the Matrix Metalloproteinases/Tissue Inhibitor of Metalloproteases Ratio and Infection as Postulated Mechanisms

Molecular biological studies suggest that angiogenesis, infiltration of inflammatory cells, and increased expressions of inflammatory cytokines such us cyclooxygenase (COX)-1, COX-2, IL-1, IL-6, and TNF- α, are present in FS. The inflammation might be the first manifestation of FS at the molecular level, and COX-1, COX-2, IL-1, IL-6, TNF-α, might play an important role in triggering, regulating, and the remission of inflammation in FS (41). A chronic state of low-grade infection/inflammation might predispose a person to develop FS (42). Inflammatory lipoproteins such as low-density lipoproteins (LDL) and non-high density lipoproteins (non-HDL), associated with vascular inflammation and immune reactions have been identified as independent risk factors for FS (23, 43). Vascular endothelial cell activation is accompanied by an increased expression of intercellular adhesion molecule-1 (ICAM-1), which has been shown to present elevated levels in the joint capsule and synovial fluid of FS patients compared to controls. Furthermore, the maintenance of chronic LGI has been shown to produce phenotypic alterations in fibroblasts and therefore lead to FS (21). This persistent fibroblast activation may play a role in the pathology of frozen shoulder and could explain the cellular mechanisms behind capsular fibrosis and persistent inflammation (21). Additionally, chronic inflammatory cells including mast cells, T and B cells and macrophages have been identified in shoulder capsule biopsies from patients with FS (44) while diseased capsule showed dysregulated cytokines such as IL-1β, IL-6 and TNF-α, which are known to drive inflammatory and matrix interactions including fibroblast activation and dysregulated collagen synthesis (45). In this context, the role of alarmins, endogenous molecules that are released into the extracellular milieu after infection or tissue injury and that signal cell and tissue damage, may play a key role, and could explain the cellular mechanisms behind capsular fibrosis and nerve growth. Alarmins high mobility group box 1 (HMGB1), IL-33, S100A8, and S100A9 have been shown to be elevated in the shoulder capsule of patients with frozen shoulder when compared to controls, as well as to be associated with significantly increased neoinnervation linked to patient-reported pain (46).

HMGB1 has been shown to be induced by damage-associated molecular patterns (DAMPs), cytokines, and certain states of cellular stress, is considered crucial to the onset and perpetuation of FS. The release of HMGB1 upon stress perpetuates the inflammatory tissue response seen in patients. The significant pain associated with frozen shoulder is linked to an increased peripheral nerve ingrowth (47).

The involvement of IL-33 has been shown to be part of the key pathologic features of angiogenesis and capsular tissue fibrosis since elevated nuclear expression of IL-33 in FS stromal cells has been found. Moreover, IL-33, when released from the resident tenocyte, has the ability to modulate inflammatory/matrix crosstalk and thus, it is likely to be important in the balance between reparation and degeneration in tissue repair.

It appears that in FS the S100 proteins are upregulated in macrophages and are therefore likely to be involved in macrophage differentiation and recruitment, which supports the concept that FS has a strong inflammatory and immunological origin (46). Under such circumstances, the extracellular matrix (ECM) that the fibroblasts produce is altered and an imbalance in ECM turnover appears, as well as an altered ratio between MMPs and tissue inhibitor of metalloproteases (TIMPs), which has been shown to be almost ten times lower in FS patients vs. healthy controls (48). The synthesis and activity of MMPs are controlled by TIMPs, cytokines, and growth factors (49). In addition, studies have concluded that mechanical stress may transduce cell signaling of mitogen-activated protein (MAP) kinase by β1-integrin to change cytokines and MMPs in the fibroblasts of FS (25).

Recently, another possible cause for the development of FS has been described: an infectious one, related to several bacteria that usually inhabit human skin. The most often identified is *Propionibacterium acnes* (*P. acnes*), an anaerobic but aerotolerant Gram-positive symbiont when it inhabits the skin (50). Although several studies have found *P. acnes* in people with FS (50, 51) a recent study is in favor of discarding this theory (52). However, the infectious cause likely remains valid, based on data from a study of 10 people with FS when they were compared to 10 patients with shoulder instability. The authors found multiple alarmins (HMGB1, IL-33, S100A8, and S100A9) in the joint capsule of the FS patient group in amounts consistent with an infection, while in the control group they found no significant increase of infection related markers (46).

*P. acnes* is an infectious bacterium probably underestimated because, being anaerobic, it requires some adapted transport system and a long incubation period (53). Its late growth in favorable environments is often misinterpreted as contamination (54). The systemic infectious potential of *P. acnes* together with *Streptococcus epidermis* (*S. epidermis*) and *Corynebacterium propinquum* (*C. propinquum*) is further supported by several findings of *P. acnes* being responsible for disc herniation (55).

*P. acnes* is associated with the highest number of low-grade infections after surgical procedures (56). Its access to the interior of the body occurs through the mouth and teeth, mainly during dental procedures and more importantly, on a daily basis during tooth brushing (57). Recent studies show how an oral dysbiosis contains microbial communities that can mediate inflammatory pathologies both locally and remotely (58).

Another bacterial infiltration route in non-operated people has been identified, namely the migration of bacteria in a dormant stage into various cells including epithelials, erythrocytes and adipocytes (59). The infiltration of these bacteria into certain tissues depends on the level of oxygen present. *P. acnes* is only able to proliferate in tissues with low pO2, such as the intervertebral disc and perhaps a shoulder that lacks movement. Currently, a clinical trial with 40 patients diagnosed with FS is underway comparing the effect of using an antibiotic with the application of prednisone (Clinical Trials.gov Identifier: NCT03479502). In our opinion, based on the data, the risk of endotoxemia as a mechanism of action linked to FS should not be dismissed.

### Current Therapies

Even though there is not a well-defined model for the medical management of FS, there is a wide spectrum of local treatments available, both surgical and non-surgical (60). Frequently, the therapeutic management of FS varies considerably among specialists based on personal experience instead of published evidence (61).

It should be noted that none of the current therapeutic options are universally accepted as the most effective in restoring symptoms in patients with FS (62).

Common nonsurgical treatments include medication, physical therapy, exercise, manipulation under anesthesia, steroid injection or nerve blockers which can provide temporary relief of symptoms (63). Most drug treatments target COX-1, COX-2 and glucocorticoid receptors with a marginal effect on inflammation and no impact on the accumulation of fibrotic collagenous tissue (64). Surgical methods include open or arthroscopic capsular release and hydrodilation, which improve the shoulder range of motion and alleviate pain but leave other complications (63).

Persistent pain and limited motion despite 3 to 6 months of conservative treatment should be taken into account in the choice of surgical treatment (65).

The treatment of frozen shoulder evolves according to the stage of the syndrome (66, 67). Thus, during the freezing phase (duration 10–36 weeks) pain is the most prominent symptom, and steroid injections are the first choice of treatment (67). In the frozen phase (4–12 months) restriction of joint movement prevails; in this case, mobilization techniques and joint distension are usually selected despite the limited evidence found (66). Finally, in the thawing phase (12–42 months) there is a reduction of pain and a progressive improvement of joint mobility. A recent systematic review and meta-analysis shows how intra-articular corticosteroid infiltrations are the prevalent intervention in the non-surgical management of FS, especially for pain control and joint function. The combination of corticosteroid infiltrations and physiotherapy may offer some improvement in the pain and freezing phase, with physiotherapy and manual mobilization offering the most benefit in the adhesive phase of the injury (68).

**Figure 1:**
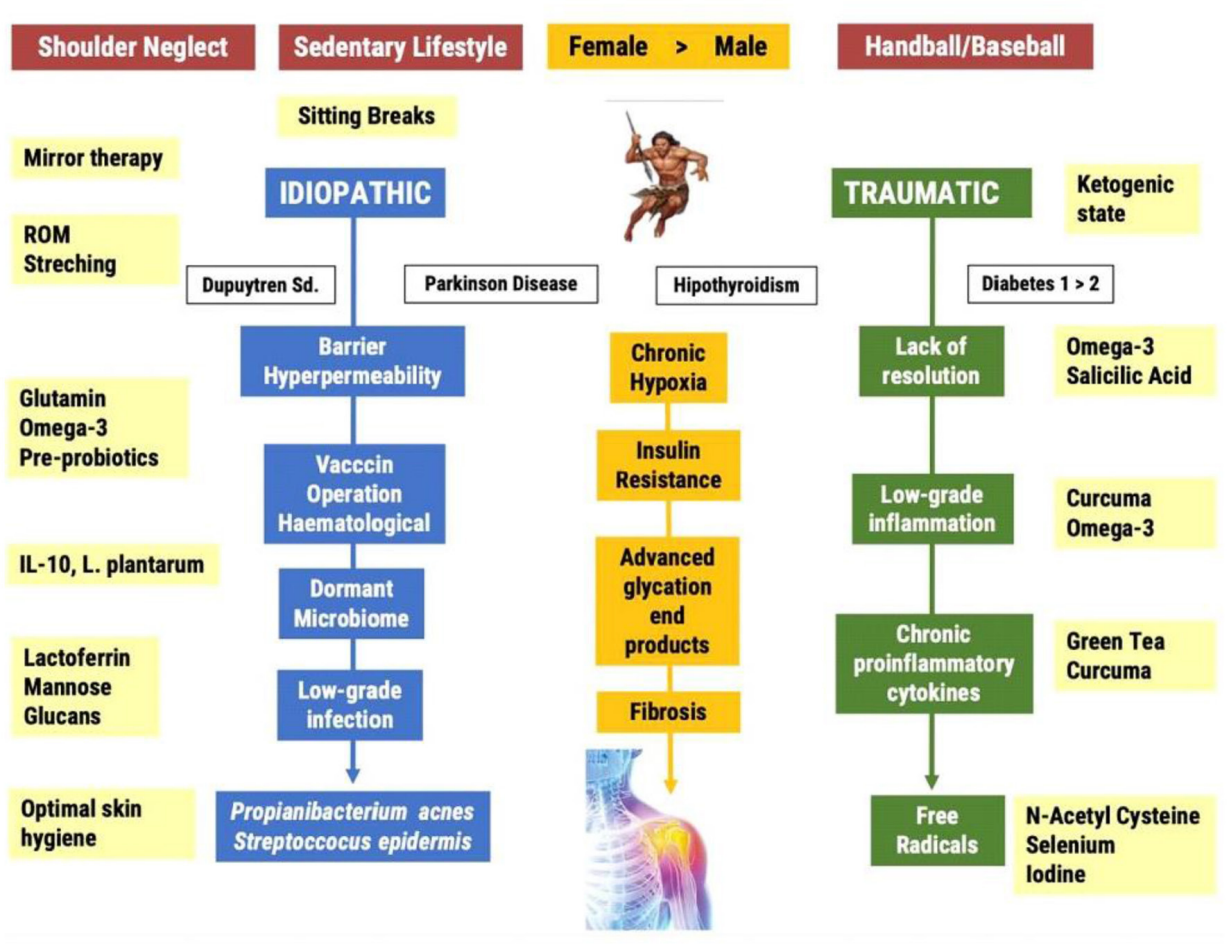
Current and proposed pathways leading to the Frozen Shoulder Syndrome; from the photo to the film.

In Figure 1 we propose an outline of the pathophysiology of FS, both at the diagnostic and therapeutic levels. In blue, we show the process associated with the “idiopathic” development of FS. In green, the process related with the development of traumatic FS, for instance by repeated throwing in sports such as baseball or handball. In yellow, we indicate the interventions aimed at the risk factors and mechanisms of action responsible for this mysterious pathology. In white, we highlight the pathologies associated with a greater risk of developing FS. The mechanisms of action discussed in this review provide a reasonable explanation for these associations. We must make clear that Dupuytren's syndrome is not the cause of frozen shoulder, but a comorbid pathology connected to the same pathophysiology behind both FS and Dupuytren, including insulin resistance, LGI and low-grade infection (46). The interventions mentioned in Figure 1 aim to offer a solution with the least number of side effects.

Frozen shoulder can be primary (idiopathic) or secondary (traumatic). Baseball and handball players often suffer complete ruptures of the rotator cuff muscles leading to surgical operations and long-term immobilization. The inability to resolve the inflammatory process promotes the establishment of a chronic LGI process in an environment of chronic hypoxia. The consequence of this state is an increased production of free radicals and the formation of fibrosis. The idiopathic route is associated with a neglect syndrome and a sedentary lifestyle. Sedentary living is the main risk factor for the development of insulin resistance and the accumulation of advanced glycation end products in the ligamentary and capsule areas of the non-dominant shoulder. The lack of voluntary movement generates chronic hypoxia and an environment which is suitable for the infiltration of a dormancy microbiome, especially anaerobic bacteria such as *P. acnes*. The low-grade infection induces the infiltration of immune cells, inflammatory cytokines, free radicals and finally the frozen shoulder.

### Proposed Interventions Based on Postulated Mechanisms

Based on the systemic nature of the postulated pathogenic mechanisms and given the limitation of publications with this approach, we propose a series of interventions aimed at the mechanisms of action described and perfectly complementary to current treatments which, in our opinion, deserve to be investigated in the field of FS.

#### Endotoxemia and Infection

Today's lifestyle is conducive to many health risks factors (69). Hormonal factors such as those associated with long-term stress, or dietary factors, either through increased consumption of foods with a high density of food additives or through the high consumption of gliadin-rich cereals pose threats to the integrity of cell-binding complexes (70). Increased permeability of body barriers is associated with a high risk of endotoxemia, chronic inflammation and insulin resistance (71). In a recent systematic review, most of the included studies showed a higher concentration of lipopolysaccharides (LPS) in diabetic patients than in healthy subjects (72).

Endotoxemia and *P. acnes* infection have been proposed as a possible mechanism linked to FS. Even though it has not been tested with patients suffering from FS, there are several *in vitro* and *in vivo* studies demonstrating lactoferrin's ability to protect against microbial infections. Lactoferrin plays an important role in several physiological processes including antimicrobial, antioxidant, anticarcinogenic, and anti-inflammatory properties (73). It exhibits inhibitory effects against several Gram-positive and Gram-negative species, including some strains that are antibiotic resistant (74). The nature of lactoferrin's mechanism of action is both, bacteriostatic and bactericidal. In a recent paper, the presence of an anti-inflammatory effect of lactoferrin on *P. acnes*-induced inflammation, both *in vitro* and *in vivo* was noticed (75). The beneficial effects of oral administration of lactoferrin have been proven in two randomized, double-blind, placebo-controlled trials (*n* = 164, *n* = 36) (76) as well as in a systematic review and meta-analysis (77).

Another intervention to be tested in order to improve endotoxemia is glutamine supplementation which may improve tissue integrity, especially in the gut, by facilitating enterocyte proliferation and the expression of tight junctions (78). In a randomized controlled study, Benjamin et al. showed how glutamine supplementation in patients with Crohn's disease in remission reduced the permeability of the gut (79). Although the results in animal models are very encouraging, human studies are still predominantly observational, unclear, and need to be standardized.

The use of prebiotics can modify the microbiota by selectively stimulating the growth and/or activity of beneficial bacteria in the gut (80). Some of the most common prebiotics are fructo-oligosaccharides (FOS) (81), which can be obtained naturally from daily consumption of vegetables, fruits and some cereals. The fiber present in these foods is specifically fermented by certain bacteria, such as bifidobacterial, among others (82). Prebiotics not only provide a source of energy for our bacteria, but also have some health benefits, such as providing both acute and chronic inflammatory relief (83).

Probiotics regulate the immune response by activating specific genes in host cells (82). Specific strains such as *Lactobacillus reuteri* (*L. reuteri*) or *Enterococcus faecium* (*E. faecium*) can increase the production of IL-10 (84). IL-10 inhibits both cytokine and inflammatory chemokine production (85). *In vitro* studies have shown that lipoteichoic acid isolated from *Lactobacillus plantarum* (*L. plantarum*) improves the inflammatory response by inhibiting the production of TNF- α and IL-6 (86). In a recent double-blind randomized placebo-controlled study, Toshimitsu T et al. demonstrated in 130 participants how the ingestion of a yogurt rich in *L. plantarum* for 12 weeks significantly improved glucose metabolism in pre-diabetic adults. They found that HbA1c values were significantly lower than in the control group. The authors stress the importance of improving this parameter since the study did not consider any other lifestyle-based intervention such as caloric restriction or increased physical activity. They suggest that using these types of probiotics optimizes HbA1c through the improvement produced in the chronic inflammatory process and insulin resistance (87).

#### Chronic Low-Grade Inflammation

We speculate that multiple signaling pathways, postulated as mechanisms belonging to FS, could be positively influenced by different compounds found in plants.

One of the alarmins considered crucial for the perpetuation of frozen shoulder is HMGB1 (46) which is released by activated macrophages/monocytes functioning as a late inflammatory mediator (88). It binds to Receptor for Advanced Glycation End Products (RAGE) which activates Mitogen-activated protein Kinases and NF-kB. Recently, several herbal medicines have been tested as HMGB1 inhibitors (89). Epigallocatechin, and epicatechin, polyphenolic compounds found in green tea (*Camellia sinensis*), have been measured *in vivo* to completely inhibit HMGB1 at a dose as low as 10μl/ml (equivalent to 75 ml/person, assuming a total body weight of 75 Kg, and blood volume of 7,500 ml) and with no measured toxicity in macrophage cultures (90). Other plants rich in polyphenolic compounds, frequently used in Chinese medicine, and with HMGB1 inhibitor properties are *Panax ginseng* (91), *Angelica sinensis* and *Salvia miltiorrhiza* (92). Further interesting polyphenolic compounds are those found in *Ecklonia cava*, a brown algae recognized as a food ingredient, and also found in the form of a supplement in Japan and Korea. Phlorotannin rich extracts found in *E. cava* attenuated serum levels of nitric oxide (NO), prostaglandins E2 (PGE2) and HMGB1in murine models (89).

The alteration of the MMPs/TIMPs ratio, which has been shown to be ten times lower in FS patients vs. healthy controls (48), might be influenced by natural substances. Three major groups of natural substances derived from plants have grown in interest because they have been shown to interfere with MMPs expression: flavonoids, green tea polyphenols and curcumin. Due to their use as food components, the risk for major toxicity and side effects seems to be very small, and epidemiologic data suggest a satisfying level of bioavailability (93).

*In vitro* studies have demonstrated that flavonoids such as genistein and nobiletin downregulate MMP-2 synthesis by inhibiting tyrosine kinases (94). Epigallo-catechin gallate (EGCG) from green tea reduces the activation of MMP-2, downregulates the MMP-2/9 expression and induces apoptosis (95). Finally, the following effects of curcumin have been shown: antiproliferative effect, induction of apoptosis by downregulation of antiapoptotic proteins, such as Bcl-2, suppression of NF-kB, inhibition of activating protein-1 (AP-1) signaling pathway, inhibition of MAPK pathway and the serine protein kinase pathway (96).

Dominiak K.et al. examined whether consumption of a formulation based on curcumin, resveratrol, EGCG and soy isoflavones could protect human lymphocytes against oxidative stress induced by *ex vivo* exposure of lymphocytes to TNF-α. This pilot study supports the use of certain natural agents in supplemental form as they may protect humans against oxidative damage and inflammation (97).

#### Chronic Pain and Loss of Mobility

IL-33 plays a pivotal role in FS in relation to pain, inflammation, angiogenesis and capsular tissue fibrosis (46). Naringenin, found in citrus, is a flavonoid with low toxicity and anti-inflammatory properties (98). In mice, Pinho-Ribeiro et al. tested the analgesic effect of this flavonoid in mechanical inflammatory pain with control groups using anti-inflammatory drugs indomethacin or dipyrone. The results suggest that naringenin inhibits both inflammatory and neurogenic inflammation. Its mechanism of action involves the inhibition of hyperalgesic cytokines (IL-33, TNF- α and IL-1β) (99).

The type of pain in FS is nociceptive as a result of all the peripheral abnormalities associated with the capsular structures. However, chronic inflammatory mediators play a role in the process of central sensitization (100). Cytokines and other local inflammatory components released by the inflamed environment may be responsible for both central and peripheral sensitization (101). Until now, central sensitization in FS has not been studied and so remains speculative, but this could explain why some patients are resistant to current interventions and may benefit from a different approach (100).

In this context, mirror therapy has proved to be effective in patients who suffered from phantom limb, complex regional pain syndrome or stroke, where central sensitization is present (102). Along the same line, two limited studies have been published regarding patients suffering from adhesive capsulitis or chronic shoulder pain with limited active range motion (AROM). Louw A. et al. measured the effects of mirror therapy in 69 patients with shoulder pain and limited AROM and found a significant increase (mean 14,5°) in affected shoulder flexion AROM, immediately after mirror therapy (*P* < 0.001). They also noticed a statistically significant improvement in pain and fear avoidance facilitating the transition to manual therapy and exercise in these patients (103). Başkaya C.T. et al. enrolled 30 patients diagnosed with adhesive capsulitis in a prospective, randomized, controlled, single-blind clinical study and found a significant increase of active and passive abduction and active and passive flexion ROM compared with the control group that received standard physical therapy. A significant improvement was noted in physical function, physical role limitation, emotional role limitation and pain parameters in the mirror group, whereas there were no significant changes in the control group (104).

Mirror therapy is an easy, non-invasive and cheap therapy consisting of the use of a mirror that reflects the movement of the unaffected part of the body, while the affected limb remains hidden (not visible). The rationale for using mirror therapy is based on the brain recovering the presence of the affected arm as being normal and as an anti-neglect intervention through visual stimulation.

A meta-analysis has measured the efficacy of using supplements rich in omega-3 fatty acids and their analgesic effect on inflammatory joint pain, highlighting how eicosapentaenoic acid (EPA) and docosahexaenoic acid (DHA) supplementation reduces the intensity of joint pain, morning stiffness and non-steroidal anti-inflammatory drugs (NSAID) consumption (105).

In patients with regional pain syndrome, Jung et al. found that diet is associated with neuroinflammatory processes and that neuroinflammation is associated with peripheral glucose levels, among other things (106). In our opinion, the potential of a ketogenic diet is worth exploring because of its ability to influence glucose metabolism, inflammation and pain. This high-fat, low-carbohydrate diet stimulates liver ketogenesis and the production of acetone, acetoacetate, and β-hydroxybutyrate (bHB) as alternative energy sources (107). The ketone bodies have functions that go beyond acting as energy resources, especially bHB, which can act on multiple levels. bHB shortens oxidative stress by inhibiting the activity of histone deacetylases class I (HDAC-I) and could further reduce the inflammatory and sympathetic system activity and increase neuroprotection through regulation of coupled G-protein receptors (GPR) and immune cells via GPR109 (108).

bHB also inhibits the NLR family, pyrin domain-containing 3 (NLRP3) inflammasome, which results in an inhibition in the production of inflammatory cytokines such as IL-1 and IL-18 (109). This pathway may be highly significant, since *P. acnes* is responsible for activating NLRP3 (110) within the pathophysiology of intervertebral disc degeneration. Both animal and human model studies have shown how a 3 to 4 weeks ketogenic diet results in both inflammatory and pain improvements (111).

## Conclusions

This study presents a systemic view on the pathogenesis of frozen shoulder with the strength of evidence-based medicine as the basis of a systemic proposal for both the diagnostic and therapeutic levels. FS is a dysfunction related to pathologies such as diabetes, Parkinson's, Dupuytren's and hypothyroidism. The mechanisms of action related to the aforementioned pathologies include low-grade inflammation and chronic hypoxia. These mechanisms, together with the influence of modern life, including a sedentary lifestyle, and the partial or complete absence of range of motion of the shoulders in general and of the non-dominant shoulder in particular, suggest that the pathology of FS is systemic. A systemic pathology requires a systemic intervention. Our proposal offers a systemic view for people afflicted with FS. Although robust evidence is still lacking, the treatment described in this review deserves to be investigated using different clinical trials. The proposed interventions do not place a large burden on the patient. FS does not seem to resolve itself, although this paradigm still persists, and more research is needed to corroborate it. The systemic approach proposed in this review could serve as the missing link to improve the success rate in people suffering from FS.

## Author Contributions

DS, FA, EL, and LP: study conception. DS, FA, EL, and LP: design. DS, FA, EL, and LP: acquisition of data. DS, SN-L, and LP: analysis and interpretation of data, drafting of manuscript, and critical revision. All authors contributed to the article and approved the submitted version.

## Conflict of Interest

The authors declare that the research was conducted in the absence of any commercial or financial relationships that could be construed as a potential conflict of interest.
